# Sexual violence in nightlife and positive bystander intervention in an English city

**DOI:** 10.1186/s12889-024-17642-7

**Published:** 2024-01-11

**Authors:** Zara Quigg, Rebecca Bates, Nadia Butler, Chloe Smith, Charley Wilson, Amanda Atkinson, Mark A Bellis

**Affiliations:** Public Health Institute, Liverpool John Moores University, 3rd Floor Exchange Station, Liverpool, L3 2ET UK

**Keywords:** Sexual, Violence, Nightlife, Alcohol, Prevention

## Abstract

**Background:**

Nightlife environments are high risk settings for sexual violence and bystander intervention programmes are being developed in response. However, more research is needed to understand nightlife-related sexual violence, and factors that influence bystander interventions. This study examined nightlife patron’s experiences of sexual violence and associated factors; and relationships between attitudes towards, awareness and experience of sexual violence, and confidence to intervene.

**Methods:**

Cross-sectional on-street survey of nightlife patrons (*N* = 307, aged 18+) on a night out in an English city. Surveys (7.30pm-1.30am; Wednesday-Saturday) established sexual violence awareness, myth acceptance, and experience, and confidence to intervene. Participant’s socio-demographics, nightlife alcohol consumption, and frequency of nightlife usage were collected.

**Results:**

58.0% had ever experienced sexual violence whilst on a night out. In adjusted analyses, sexual violence was higher amongst females (adjusted odds ratio [AOR] 4.0; *p* < 0.001), and regular nightlife patrons (AOR 2.1; *p* < 0.05). The majority agreed that they would feel confident asking someone who has experienced sexual violence if they are okay/would like support (92.2%). In adjusted analyses, confidence to intervene was higher amongst those who agreed that sexual violence was an issue in nightlife (AOR 3.6; *p* < 0.05), however it reduced as sexual violence myth acceptance increased (AOR 0.5; *p* < 0.05).

**Conclusion:**

Sexual violence is a pertinent issue in nightlife. Programmes aiming to address nightlife-related sexual violence must address the wider social norms that promote sexual violence, and ensure patrons understand the extent and significance of the issue, to increase confidence to positively intervene.

**Supplementary Information:**

The online version contains supplementary material available at 10.1186/s12889-024-17642-7.

## Background

Over the past two decades, increasing attention has been drawn to preventing and responding to sexual violence in both domestic and public spaces [1, 2, 3]. Sexual violence is a significant public health, human rights and gender equality issue that has damaging effects on individual health and wellbeing, local communities, public services and wider society [1, 3]. Evidence shows that experience of sexual violence is unequal with women and girls at greatest risk, often rooted in unequal gender norms [1, 3, 4]. More recently, increased attention to addressing sexual violence and gender inequality more broadly has been observed in media and public discourse, in response to global social movements aimed at raising awareness of sexual harassment and violence such the 2017 Women’s March, #MeToo and #EverySexism [5, 6, 7]. Whilst this suggests a supportive social and cultural context in which prevention approaches are now situated, gaps in evidence exist, and understanding the nature and extent of sexual violence, its root causes, and factors that can prevent harm, is critical for informing prevention activity. Various factors increase the risk of victimisation and perpetration, and whilst sexual violence occurs in both private and public spaces, across various countries nightlife settings have been identified as ‘hot spots’ where sexual violence is both highly prevalent, and socially accepted [3, 4, 8, 9].

Nightlife is a transgressive environment in which pleasure, intoxication and sexual expression and relations (i.e., a site to find sexual partners) co-exist [3, 9, 10, 11]. Whilst for many, nightlife is a safe space for pleasure and fun [12], safety can be compromised, particularly for women, due to the prevalence of risky behaviours (e.g. alcohol/drug use; sexual risk taking), and social norms that render sexual violence more acceptable and expected than in other aspects of everyday life [3, 4, 8, 9, 11, 13, 14, 15]. This includes perceived associations between alcohol use and sexual availability (e.g., that drunk women are ‘loose’), hyper sexualisation and the normalisation of sexualised dress that can be misread as a sign of sexual availability, and an acceptance of some forms of sexual touching (i.e. bottom grabbing) [3, 4, 11, 13, 16]. Across several countries, studies have shown that lifetime prevalence of nightlife-related sexual violence amongst patrons can reach over 50%^3^. Critically, a wide range of risk factors at an individual, relationship, and community/environmental level have been shown to increase risks of sexual violence in nightlife [3]. For example, women, and younger people have been identified as more at risk of victimisation, and several studies highlight associations between nightlife-related sexual violence and increased alcohol consumption, signs of intoxication and engagement in specific drinking patterns by either the victim or perpetrator [3].

Whilst rates differ, sexual violence is a normalised aspect of nights out for many women globally [3, 10, 17]. Subsequently and importantly, efforts to prevent nightlife-related sexual violence are developing with pace, and critically there is an increasing focus on challenging the social norms that support sexual violence and increasing positive bystander intervention [3, 18, 19]. Across the United Kingdom, Europe and elsewhere (e.g., Australia, USA), interventions to promote nightlife workers positive bystander intervention are emerging. These differ from individualistic approaches that focus on encouraging the use of risk reduction techniques among potential victims/survivors, and approaches that focus on perpetration. The former has been shown to potentially generate backlash due to responsibility being placed onto the potential victim, and the latter defensiveness among men [11, 18, 20, 21]. Instead, bystander intervention takes a socio-ecological approach that views sexual violence and the social norms that underpin it as a public health problem, for which the causes are complex and occur at the individual, group, community and societal level. Critically they aim to shift prevention responsibility from the individual to the community [3, 18, 22]. Related to social norms theory, the aim is to change the social and cultural norms that exist in specific environments such as nightlife, which condone and support sexual violence, and remove barriers to intervening [18, 23, 24]. For example, to be an effective bystander who can positively intervene, bystanders need to be aware of sexual violence and recognise it as a problem, and have the sense of responsibility and necessary skills to intervene [25]. All participants in a given environment or community (e.g., University campus, workplace, nightlife) are regarded as bystanders (i.e., third party witnesses) to the norms that promote sexual violence, and as such are viewed as having a role and responsibility (i.e., as an ‘allie’ in helping others) in shifting these social norms and preventing sexual violence [21, 22]. Intervening is encouraged by increasing awareness of violence, changing attitudes to better foster a responsibility to intervene, and building skills and knowledge of the tactics on how to do so safely and effectively [20].

A number of individual and situational factors influence the likelihood of a bystander intervening (i.e. becoming a ‘responsive bystander’) [21], and include their level of rape/sexual violence myth acceptance (e.g. if people are drunk or dress a certain way they are responsible for their experiences) [18, 20, 24], relationship to the victim and perpetrator [11], the gender of the bystander and victim [11, 26], and bystander efficacy (e.g. confidence to intervene) [20]. Promisingly, recent pilot evaluations suggest that bystander interventions reduce rape myth acceptance and increase willingness to intervene among nightlife workers [18, 19, 27]. Despite these positive indications, there remains a scarcity of research regarding factors that may mediate positive bystander intervention in this context, and particularly amongst nightlife patrons. To inform the development of such interventions, it is vital to understand nightlife patron’s experiences and social norms in relation to sexual violence, and how this may influence positive bystander behaviours. Whilst studies across various countries illustrate the nature and extent of nightlife-related sexual violence and norms that support this [3, 9], to the best of our knowledge, no study has explored associations with bystander behaviour amongst nightlife patrons. Thus, our study aimed to explore:

1. The nature and extent of sexual violence among nightlife patrons; and factors associated with victimisation.
2. How experience of, and attitudes towards and awareness of sexual violence, influence confidence to positively intervene in sexual violence.

## Methods

### Study design, participant recruitment and sample

A quantitative survey captured participants’ experiences of sexual violence whilst on a night out, and concepts relating to bystander behaviour including awareness of sexual violence and myth acceptance, and confidence to act as a positive bystander to respond to sexual violence. The research took place within a large nightlife area (~ 250 on-licensed premises) of one city in the North of England, popular among locals, students and international tourists for its nightlife. The area in which the data was collected is a well-known predominantly heteronormative mainstream area that consists of a mix of independently owned and chain bar establishments.

All the researchers attended training prior to the commencement of fieldwork and were briefed on researcher safety and ethical protocol concerning informed consent. A team of male and female researchers approached potential participants in public spaces outside nightlife venues between the hours of 7.30pm-1.30am over four nights aligning with peak nightlife activity and enabling breaks between field researcher activity (week 1: Wednesday and Friday; week two: Thursday and Saturday; June 2022). Before approaching potential participants, researchers conducted a visual assessment of their intoxication level based on criteria used by the police and in previous studies that involved fieldwork in nightlife environments [28]. This included assessing for signs of unsteadiness (on their feet) and loud or aggressive talking (and upon direct contact signs such as difficulty focusing, slurring words/incoherent speech, glazed eyes).

Researchers did not approach nightlife patrons who appeared to be highly intoxicated for safety reasons and concerns that if a person was highly intoxicated they may not be able to give informed consent. Researchers confirmed that the potential participant was over the age of 18 years and explained the nature of the study and assured them of the anonymity of their responses. If they agreed to take part, participants were handed an information sheet, which confirmed the study details and provided information of local support services for those that had queries or concerns relating to sexual violence. Out of the 812 potential participants approached, 324 agreed to participate (response rate 39.9%). Surveys were completed on a tablet, with questions read out and answers recorded by the researcher (to enhance confidentially, participants could point to answers, and had the option to self-complete). Only those with complete data to determine if they had or had not experienced sexual violence were included in the study (*n* = 307; those with missing data had typically ended their participation in the study prior to survey completion, as they wanted to progress with their night out). 54.8% of surveys were completed on the Wednesday/Thursday, and 58.2% between the hours of 22.30pm and 01:30am. Ethical approval for the study was granted by Liverpool John Moores University Research Ethics Committee.

### Measures

#### Experience of sexual violence

Respondents were asked if they had ever personally experienced sexual violence when on a night out in the nightlife setting under study (including during and after a night out, e.g., at home). To aide understanding of sexual violence, they were provided with a short description– ‘sexual violence is the general term we use to describe any kind of unwanted sexual act or activity, including rape, sexual assault, sexual abuse, harassment, unwanted sexual attention such as cat calling and many others’. Response options included yes (in the past 12 months / not in the past 12 months), no or prefer not to say. All reporting yes where subsequently asked if they would be happy to answer questions about their most recent experience, enabling participants to opt-out of further detailed questions to reduce risks of re-traumatisation (6% of respondents opted out at this stage). Additional questions covered the nature and location of the incident, relationship to, and gender of the perpetrator, and whether they reported the incident to anyone.

#### Awareness of sexual violence

Respondents were asked to rate how much they agreed (1 = strongly agree to 5, strongly disagree) that ‘sexual violence is a problem in nightlife’ (one item, adapted from existing validated scales, and used in previous nightlife research) [19, 27].

#### Sexual violence myth acceptance

Respondents were asked to rate how much they agreed (1 = strongly agree to 5, strongly disagree) with five items relating to sexual violence myth acceptance: (1) if someone who is experiencing sexual violence is drunk, they are at least partly to blame; (2) if the person committing sexual violence is drunk, it is not really their fault; (3) sexual violence is never the fault of the victim; (4) a women should be able to wear what she wants without being at risk of sexual violence; and, (5) consent can be taken back at any time. Items were adapted from existing validated scales and have been used in previous nightlife research [19, 27]. An overall score for sexual violence myth acceptance was developed based on the mean of the items (with items 1 and 2 scores reversed prior to inclusion in the combined score). Cronbach’s Alpha for the five items was 0.48.

#### Confidence to act as a positive bystander

Respondents were asked to rate how much they agreed (1 = strongly agree to 5, strongly disagree) with six items examining their confidence to positively intervene as a bystander to sexual violence. This included confidence to ask someone who has experienced sexual violence if they are okay and if they would like further support (adapted from existing validated scales and used in previous nightlife research) [19, 27]; and confidence to ask others (i.e., friend, nightlife worker, transport worker, police officer and a stranger) for help to intervene in sexual violence. Our measure for bystander intervention used in this study utilised one item: confidence to ask someone who has experienced sexual violence if they are okay and if they would like further support (referred to as confidence to intervene in sexual violence).

#### Demographic and social characteristics

The following variables were included in univariate and multi-variate analyses based on existing evidence on risk factors for nightlife-related sexual violence: sex, age, sexuality, and alcohol consumption on a typical night out (see Table 1) ^3^. Frequency of visiting the nightlife setting was included to account for increased exposure to the environment where sexual violence is reported as often prevalent [3].

**Table 1:** Univariate and multi-variate analyses showing associations with experience of sexual violence (SV)

		Never experienced SV% (n)	Have experienced SV % (n)	OR (95% CI)	P	AOR (95% CI)	P	
	Total	42.0% (129)	58.0% (178)					
Sex	Female	31.8% (69)	68.2% (148)	4.4 (2.6–7.4)	< 0.001	4.0 (2.6–7.1)	< 0.001	
	Male	67.0% (59)	33.0% (29)			1.0		
Age group (years)	18–21	30.9% (43)	69.1% (96)	3.8 (1.9–7.4)	< 0.001	2.1 (1.0-4.2)	0.051	
	22–29	46.0% (52)	54.0% (61)	2.0 (1.0-3.9)	< 0.05	2.0 (1.0–4.0)	0.061	
	30+	63.0% (34)	37.0% (20)			1.0		
Sexuality	Heterosexual	45.6% (109)	54.4% (130)	0.5 (0.3–0.9)	0.018	0.6 (0.3–1.2)	0.136	
	Other ^c^	29.4% (20)	70.6% (48)			1.0		
Regular nightlife user ^a^	Yes	28.1% (25)	71.9% (64)	2.4 (1.4–4.1)	< 0.05	2.1 (1.1–3.7)	< 0.05	
	No	48.1% (104)	51.9% (112)			1.0		
Regular nightlife drinker ^b^	Yes	39.6% (91)	60.4% (139)	1.5 (0.9–2.5)	0.138	NA	NA	
	No	49.3% (37)	50.7% (38)					

^a^ Go out in the NTE at least once a week ^b^ Drink alcohol every time or almost every time they visit the NTE ^c^ Lesbian/gay, bisexual/pansexual, queer, other/prefer own term

### Analyses

SPSS was used to undertake all analyses. To examine factors associated with lifetime experience of sexual violence (question 1), univariate analyses examined associations with demographics and social characteristics; significant variables identified at this level were subsequently included in multi-variate analyses (logistic regression). To examine factors associated with confidence to intervene in sexual violence (question 2), univariate analyses examined associations with demographics and social characteristics, experience of sexual violence (lifetime), sexual violence myth acceptance and awareness of sexual violence; significant variables identified at this level were subsequently included in multi-variate analyses (logistic regression).

## Results

### Demographics and alcohol use

The majority (71.1%) of participants were female, aged 18–21 years (45.3%) and identified as heterosexual/straight (77.9%) (see Supplementary Table 1). Over a quarter (29.2%) indicated that they typically go on a night out in the nightlife area at least once a week. 75.4% reported that they drink alcohol every time or almost every time they visited the nightlife area in the past 12 months. Of those, 38.5% reported preloading alcohol every time/almost every time, and 41.1% reported typically drinking 10 + standard drinks over the course of the night out (including preloading).

### Experience of sexual violence

Over half (58.0%) of all participants (*n* = 307) reported having ever experienced sexual violence whilst on a night out in the nightlife area; 44.0% reported that they had experienced sexual violence in the past 12 months. In univariate analyses, there were significant associations between experience of sexual violence and sex, age group, sexuality and regularity of nightlife use (Table 1; *p* < 0.05). In multi-variate analyses, only sex and regularity of nightlife use remained significant (Table 1). Females were 4.0 times more likely to experience sexual violence in nightlife than males (*p* < 0.001), and regular nightlife patrons 2.1 times more likely than non-regular nightlife patrons (*p* < 0.05).

The majority (94.4%; *n* = 168) of those who had ever experienced sexual violence agreed to answer further questions about their most recent experience. Of these, 51.2% reported that the incident included physical and verbal violence, 27.4% physical only, and 19.6% verbal only. The majority (78.0%) reported that it occurred in a pub, bar or nightclub, 39.8% in the street or public setting, 13.9% on transport or around transport pick up points, 4.8% in a food establishment and 3.6% in another location. The majority (86.9%) reported that the perpetrator was a stranger; 9.1%, 6.1% and 1.2% reported it being an acquaintance, a friend and a nightlife worker respectively (participants could pick more than one option). Nearly all (91.6%) reported that the perpetrator was a male (women 97.9%; men, 54.2%); 7.2% selected female (women 0.7%; men, 44.0%). Two-thirds (69.0%) stated that they did not report the incident to anyone. A quarter (24.1%) reported the incident to a friend or family member; with less than one in twenty reporting it to the police (4.6%), a door supervision or security staff member (4.0%), bar staff (2.9%), health practitioner (1.1%) or welfare officer (1.1%).

### Attitudes towards and awareness of sexual violence, and confidence to intervene

The majority (92.2%) of participants agreed that sexual violence was an issue in nightlife. The mean score for sexual violence myth acceptance was 1.5 (range 1-3.6; higher scores indicate greater agreement with sexual violence myths). The majority (92.2%) of participants agreed that they would feel confident asking someone who has experienced sexual violence if they are okay and if they would like further support (i.e., our measure for bystander intervention - confidence to intervene in sexual violence). The majority (95.1%) also agreed that they would feel confident asking a friend for help to intervene in sexual violence; fewer agreed that they would feel confident asking a police officer (62.9%), nightlife worker (52.1%), stranger (29.3%) or transport worker (28.3%).

In univariate analyses, confidence to intervene in sexual violence was higher amongst females (*p* < 0.05); those who had experienced sexual violence (*p* < 0.05); and those who agreed sexual violence was an issue in nightlife (*p* < 0.01; Supplementary Table 2). Sexual violence myth acceptance was lower amongst those who agreed that they would feel confident asking someone who has experienced sexual violence if they are okay and if they would like further support (mean 1.43 cv. 1.83; *p* < 0.01). In multi-variate analyses, confidence to intervene was associated with awareness of sexual violence and sexual violence myth acceptance (Table 2). Those who viewed sexual violence as a problem in nightlife were 3.6 times more likely to have the confidence to intervene (*p* < 0.05). However, as agreement with sexual violence myths increased, the odds of having the confidence to intervene decreased (adjusted odds ratio 0.5, *p* < 0.05).

**Table 2:** Multi-variate analysis showing associations with confidence to intervene in sexual violence

Factor		AOR (95% CI)	p	
Sex	Female	2.2 (0.9–5.6)	0.094	
	Male	1.0		
Experienced sexual violence	Yes	1.7 (0.6–4.4)	0.313	
	No	1.0		
Agree sexual violence is a problem in nightlife	Yes	3.6 (1.2–11.4)	0.027	
	No	1.0		
Sexual violence myth acceptance	Mean	0.5 (0.2–0.9)	0.024	

AOR = Adjusted odds ratio; CI = confidence interval

## Discussion

Like past research, the study found a high prevalence of sexual violence victimisation on nights out, particularly among women and those who more regularly frequent nightlife [3]. Promisingly, most reported feeling confident asking someone they had witnessed experiencing sexual violence if they are okay/would like support. However, sexual violence awareness and myth acceptance acted as barriers to bystander intervention, in that being less aware of sexual violence as a problem in nightlife, and holding views that supported myths, was associated with feeling less confident intervening.

Our study demonstrates how pervasive sexual violence victimisation is amongst nightlife patrons when visiting the night-time economy. Further, it highlights the context of experiencing sexual violence which may help inform prevention activity. For example, incidents primarily occurred inside pubs, bars and nightclubs, and whilst most were perpetrated by strangers, a proportion were perpetrated by an acquaintance or friend. Critically, over two-thirds of victims stated that they did not report their most recent incident to anyone, and amongst those who did this was typically a friend or family member rather than someone with authority, who may provide additional support (e.g. police or nightlife worker). This raises the importance of implementing research to understand nightlife-related sexual violence, and to enable societal shifts to address factors that promote sexual violence and underreporting. Due to the in-person nature of the survey in a public setting, to limit the potential of safeguarding risks to the participant (and to avoid retraumatisation) we did not ask about the specifics of sexual violence experience such as severity or types of sexual violence (beyond physical or verbal abuse). Whilst this may be useful for the development and implementation of prevention approaches, the research adds more broadly to the growing evidence base highlighting the critical need to implement interventions to prevent sexual violence in nightlife settings. Programmes should aim to raise awareness of the prevalence and impact of sexual violence as an important issue within nightlife and also address the social norms that may underpin the normalisation of sexual violence, including sexual violence myths.

Previous evaluations show that bystander intervention programmes are effective in reducing sexual violence myth acceptance among nightlife workers (e.g. bar staff), as well as increasing a willingness to intervene [18, 19, 27]. Furthermore, evidence on implementation of sexual violence programmes in college campuses suggests they can be effective in preventing sexual violence [20, 21, 29]. Whilst our study suggests that nightlife patrons are likely to be willing to intervene in sexual violence, due to the complexities of nightlife, further research is needed to examine the transferability of bystander programmes to target nightlife patrons. Bystander intervention programmes are likely to require adaptation to appropriately target nightlife patrons (who may be intoxicated) and the nightlife setting (which is often dark, busy, and frequented by different people throughout the night). Importantly, programmes must consider other environmental factors that may undermine their success, including the sexualised nature of nightlife itself, and the way venues are marketed through hyper sexualised and objectifying images of women that reinforce social norms that condone sexual violence, and the role of alcohol on patron’s ability to notice and/or intervene in sexual violence [3, 9, 10, 30, 31].

Of concern is how despite the majority of participants reporting feeling confident in intervening as a bystander, and in requesting support from others in doing so, the majority of those who had experienced sexual violence themselves had not reported the incident(s) to anyone. Work is required to support victims/survivors to access support if required. In light of survey research [32] that found 75% of UK adults felt a need for improved safety procedures in bars, pubs and nightclubs to address the safety of women and girls, there appears to be public demand and support for action on addressing nightlife-related sexual violence. The research provides evidence to suggest that this should include interventions that raise awareness of sexual violence and reporting processes, and that address sexual violence myth acceptance among nightlife patrons.

### Limitations

Several study limitations should be acknowledged. Firstly, the cross-sectional nature of the survey prohibits any causal inferences being made. Secondly, the study did not generate data to address the extent to which people did actually intervene but explored intent. Further, whilst our survey items were informed by validated scales, due to survey length we did not use a full validated scale to measure bystander attitudes or behaviours and the Cronbach’s alpha for sexual violence myth acceptance was low (0.48; removal of items did not improve this). Future studies should consider this. Next, there may have been a self-selection bias with those who considered sexual violence to be a problem or having experienced it themselves, agreeing to take part. Our response rate was 43.8%, which is higher than a comparable nightlife patron study on sexual violence (Canada, 16% [13]), yet slightly lower than yet more comparable to a study examining nightlife patron behaviours (United Kingdom, 50.6% [28]). Due to the complexities of surveying nightlife patrons, the study did not use a random sampling approach and thus the findings may not be representative. Last, the research was conducted in a heteronormative nightlife setting, and as such captured the experiences and perceptions of mainly heterosexual people (77.9%). It is important that future research explores experiences of sexual violence among minority genders and sexualities [33]. Intersectionality is important to incidences of sexual violence, and research and programmes must consider how different identities (i.e., genders, sexualities, ethnicities) may have different experiences and attitudes that influence their readiness and confidence to intervene.

## Conclusion

Sexual violence is a pertinent issue in nightlife, particularly for women. The findings support existing bystander intervention theory, that emphasises a need to increase awareness of the problem of sexual violence in nightlife, and address sexual violence myth acceptance, in order to increase confidence, and in turn, positive bystander interventions. Collaborations between a range of stakeholders are needed to effectively implement and evaluate sexual violence prevention programmes. Specific consideration should be given to the development, implementation and evaluation of sexual violence bystander programmes as part of a suite of programmes to prevent such harms in the night-time economy.

### Electronic supplementary material

Below is the link to the electronic supplementary material.

Supplementary Material 1

## Data Availability

The data generated and analysed in the current study is available from the corresponding author on reasonable request.
