# Supplementary material for: Sexual violence in nightlife and positive bystander intervention in an English city
Source: BMC Public Health. 2024 Jan 11;24:157. doi: 10.1186/s12889-024-17642-7 (PMC10782665; doi:10.1186/s12889-024-17642-7)
Supplement: Supplementary file 1 — Supplementary Material 1 [file 12889_2024_17642_MOESM1_ESM.docx]

**Additional file 1**

**Supplementary Table 1: Sample characteristics**

|  |  | % (n) |
| --- | --- | --- |
|  | Total | 307 |
| Sex | Female | 71.1% (217) |
|  | Male | 28.9% (88) |
| Age group (years) | 18-21 | 45.3% (139) |
|  | 22-29 | 36.8% (113) |
|  | 30+ | 17.6% (54) |
| Sexuality | Heterosexual | 77.9% (239) |
|  | Other ^c^ | 22.1% (68) |
| Regular nightlife user ^a^ | Yes | 29.2% (89) |
|  | No | 70.8% (216) |
| Regular nightlife drinker ^b^ | Yes | 75.4% (230) |
|  | No | 24.6% (75) |
| ^a^ Go out in the NTE at least once a week ^b^ Drink alcohol every time or almost every time they visit the NTE ^c^ Lesbian/gay, bisexual/pansexual, queer, other/prefer own term. | | |

**Supplementary Table 2: Univariate analyses showing associations with confidence to intervene in sexual violence**

|  |  | Confident to intervene % (n) | OR (95% CI) | p |  |
| --- | --- | --- | --- | --- | --- |
|  |  |  |  |  |  |
|  | All | 92.2% (283) |  |  |  |
| Sex | Female | 94.5% (205) | 2.7 (1.2-6.3) | 0.021 |  |
|  | Male | 86.4% (76) |  |  |  |
| Age group (years) | 18-21 | 97.1% (135) | 2.7 (0.7-11.2) | 0.171 |  |
|  | 22-29 | 85.8% (97) | 0.5 (0.2-1.5) | 0.217 |  |
|  | 30+ | 92.6% (50) |  |  |  |
| Sexuality | Heterosexual | 91.6% (219) | 0.7 (0.2-2.1) | 0.503 |  |
|  | Other ^c^ | 94.1% (64) |  |  |  |
| Regular nightlife user ^a^ | Yes | 96.6% (86) | 3.0 (0.8-10.1) | 0.09 |  |
|  | No | 90.7% (196) |  |  |  |
| Regular nightlife drinker ^b^ | Yes | 92.6% (213) | 1.3 (0.5-3.2) | 0.588 |  |
|  | No | 90.7% (68) |  |  |  |
| Experience of sexual violence | Yes | 95.5% (170) | 3.0 (1.2-7.3) | 0.014 |  |
|  | No | 87.6% (113) |  |  |  |
| Sexual violence is a problem in nightlife | Yes | 93.6% (265) | 4.9 (1.7-13.9) | 0.003 |  |
|  | No | 75.0% (18) |  |  |  |
| Sexual violence myth acceptance | Mean - confident to intervene | 1.43 | 0.3 (0.2-0.7) | 0.002 |  |
|  | Mean - not confident to intervene | 1.83 |  |  |  |

OR = odds ratio. a Go out in the NTE at least once a week b Drink alcohol every time or almost every time they visit the NTE c Lesbian/gay, bisexual/pansexual, queer, other/prefer own term.
